# Breast Milk Micronutrients and Infant Neurodevelopmental Outcomes: A Systematic Review

**DOI:** 10.3390/nu13113848

**Published:** 2021-10-28

**Authors:** Francesca Lockyer, Samantha McCann, Sophie E. Moore

**Affiliations:** 1 Department of Women and Children’s Health, King’s College London, London SE1 7EH, UK; samantha.mccann@kcl.ac.uk (S.M.); sophie.moore@kcl.ac.uk (S.E.M.); 2 Medical Research Council Unit, The Gambia at the London School of Hygiene and Tropical Medicine, Fajara, Banjul P.O. Box 273, The Gambia

**Keywords:** micronutrients, nutrition, breastfeeding, milk, human, infants, neurodevelopment

## Abstract

Micronutrients are fundamental for healthy brain development and deficiencies during early development can have a severe and lasting impact on cognitive outcomes. Evidence indicates that undernourished lactating individuals may produce breast milk containing lower concentrations of certain vitamins and minerals. Exclusively breastfed infants born to mothers deficient in micronutrients may therefore be at risk of micronutrient deficiencies, with potential implications for neurodevelopment. This systematic review aims to consider current knowledge on the effects of breast milk micronutrients on the developmental outcomes of infants. The databases Medline, Global Health, PsychInfo, Open Grey, and the Web of Science were searched for papers published before February 2021. Studies were included if they measured micronutrients in breast milk and their association with the neurodevelopmental outcomes of exclusively breastfed infants. Also, randomised control trials investigating neurocognitive outcomes following maternal supplementation during lactation were sought. From 5477 initial results, three observational studies were eligible for inclusion. These investigated associations between breast milk levels of vitamin B6, carotenoids, or selenium and infant development. Results presented suggest that pyroxidal, β-carotene, and lycopene are associated with infant neurodevelopmental outcomes. Limited eligible literature and heterogeneity between included papers prevented quantitative synthesis. Insufficient evidence was identified, precluding any conclusions on the relationship between breast milk micronutrients and infant developmental outcomes. Further, the evidence available was limited by a high risk of bias. This highlights the need for further research in this area to understand the long-term influence of micronutrients in breast milk, the role of other breast milk micronutrients in infant neurodevelopmental outcomes, and the impact of possible lactational interventions.

## 1. Introduction

It is now widely acknowledged that the first 1000 days of life, spanning conception to two years postpartum, constitutes a unique period of importance in infant development [1,2]. This phase coincides with the rapid growth and expansion of the infant central nervous system (CNS) [3], conferring a particular sensitivity of neurodevelopmental processes to environmental influences [1,2]. The anatomical architecture of the brain is predominantly determined at this time [4], mediated by the complex interplay between the infant’s genetic framework and their surrounding environment [2]. Therefore, experience of adversities during early infancy, such as undernutrition, can ultimately have lifelong consequences for neurocognitive outcomes [5,6].

Adequate nutrition during brain formation is vital to sustaining the normal development of infant behaviour, cognition, and socioemotional outcomes [2]. Both macronutrients (carbohydrates, lipids, and proteins) and micronutrients (vitamins and minerals which support normal bodily processes [7]) are essential in sustaining the metabolic demands of this process and contribute directly to the maturation of the CNS [1]. For example, the energy required for neurodevelopment is maintained by glucose, iron, copper [8], zinc, and selenium [1]. The structure and composition of the brain are influenced by protein and long-chain polyunsaturated fatty acid (LCPUFA) deposits, and folate via neurulation [8]. The process of neural cell differentiation is supported by micronutrients such as iodine [9], and zinc [10]. Additionally, myelination is affected by iron, copper, iodine, vitamin B12, choline [8], and cholesterol [11]. Postnatally, in exclusively breastfed (EBF) infants, breast milk (BM) provides the sole source of both macro- and micronutrients.

Nutritional deficiencies in the first 1000 days of life can therefore be detrimental to neurodevelopment; macronutrient deficiencies in early childhood are associated with compromised intellect and behaviour, which can persist into adolescence [12]. Micronutrient deficiencies during infancy can also impede later neurocognitive functioning: neonatal iodine deficiency has been linked to compromised mental ability, and in extreme cases, cretinism [13], whilst inadequate iron intake during early development can irreversibly impair behavioural outcomes [14]. Approximately two billion people globally suffer from micronutrient deficiencies [15], which contributes significantly to worldwide morbidity and mortality [7]. Micronutrient deficiencies predominantly occur in low- and middle-income countries (LMICs), and among impoverished populations, although there is also a broader risk across vulnerable groups globally [7]. Usually, deficiencies occur as a result of inadequate dietary intake, compromised absorption in the gastrointestinal tract, or during periods when the physiological demand for micronutrients is increased, for example in pregnancy, lactation, and infancy. Among infants, micronutrient deficiencies are often secondary to maternal deficiency pre- or postnatally [7,16].

Mode of feeding also influences infant neurodevelopment, as suggested by the association between breastfeeding and prolonged benefits for intellectual performance [17]. BM intake has been linked to increased white and grey matter development [18], and improved memory and motor skills in children at seven years of age [19]. Studies have also identified higher intelligence quotient (IQ) scores among individuals who have been breastfed [17], consistent after controlling for maternal intelligence, a significant confounding variable [20]. The benefits of this mode of infant feeding for developmental outcomes have been attributed to the environmental factors associated with breastfeeding, such as positive parenting practices [21], or the enhanced bond between mother and infant [22]. Additionally, evidence suggests that the constituents of BM itself contribute to neurodevelopment; one study identified a positive, dose-response relationship between the intake of BM in preterm infants and their childhood IQ, which was consistent among those receiving BM via a nasogastric tube [23].

Research has yet to ascertain which elements of BM enhance cognition, although macronutrients have been at the forefront of hypotheses. For instance, the abundance of cholesterol in human milk has been postulated to contribute to infant cognitive outcomes [24], since cholesterol is central to the formation of myelin [11,25]. Furthermore, improved neurodevelopmental outcomes linked to breastfeeding may be attributable to the LCPUFAs contained in BM. For example, docosahexaenoic acid (DHA) is a LCPUFA that accumulates in vast quantities in the brains of breastfed infants [26]. Considering the importance of micronutrients in brain development [7], it is plausible that their presence in BM also mediates infant neurocognitive function.

As indicated above, inadequate micronutrient supply during early infancy can have severe and irreversible consequences for neurodevelopment [2]. Although some micronutrients such as iron are predominantly obtained by exclusively breastfeeding infants during gestation [27], infants are more heavily dependent on BM for the provision of other vitamins and minerals, for example, vitamin B1 [28]. Exclusive breastfeeding is recommended for the first six months of life by the World Health Organization (WHO) [29], emphasising the importance of BM as a source of such micronutrients in the early postpartum period alongside the other well recognised benefits of BM. However, the micronutrient composition of human milk is highly varied across populations and may be sensitive to the dietary intake of mothers [30]. If the availability of a vitamin or mineral in BM is closely associated with maternal levels, for example in the case of BM iodine [31], and infants do not accumulate these throughout gestation, EBF infants of micronutrient depleted mothers are also at high risk of micronutrient deficiency [27]. Evidence supports this notion, indicative that lactating mothers with low levels of certain micronutrients, such as vitamin A [32], tend to have concurrently deficient infants [32,33]. For these reasons, BM micronutrients of concern are vitamin B1, vitamin B2, vitamin B6, vitamin B12, vitamin A, iodine, and selenium [28].

If BM micronutrients contribute to the beneficial impact of exclusive breastfeeding on infant brain development, then EBF infants of undernourished mothers may not reap the neurodevelopmental benefits of exclusive breastfeeding to the same extent as the infants of well-nourished mothers. In addition, the lack of evidence surrounding the supplementation of infants under six months of age further limits the formation of policy and interventions to protect these infants [34]. No currently published or pre-registered reviews have comprehensively examined the relationship between BM micronutrients and infant neurocognitive outcomes. This study aims to systematically review and synthesise the available literature relating to BM micronutrients and developmental outcomes, to further inform policy and practice surrounding exclusive breastfeeding in populations at risk of micronutrient deficiencies, and aid in understanding how exclusive breastfeeding influences infant neurodevelopment.

## 2. Materials and Methods

### 2.1. Protocol and Registration

The protocol for this review was submitted for registration on the 22nd of February 2021, at the International Prospective Register of Systematic Reviews (PROSPERO), registration number: CRD42021238435. This can be viewed at the following link: https://www.crd.york.ac.uk/prospero/display_record.php?ID=CRD42021238435 (accessed on 15 September 2021).

This review was written in accordance with the Preferred Reporting Items for Systematic Reviews and Meta-Analysis (PRISMA) guidelines [35], the checklist for which is included in the Supplementary Material (Table S1).

### 2.2. Eligibility Criteria

The review included studies measuring an association between maternal BM micronutrients (among mothers of exclusively breastfed infants) and offspring neurocognitive outcomes during, or at any time after, the period of exclusive breastfeeding. Additionally, intervention studies measuring the developmental outcomes of EBF infants exposed to maternal lactational micronutrient supplementation were eligible for inclusion. The controls in intervention studies included EBF infants of mothers who were not supplemented during lactation. Outcomes included any measurement of infant or child neurocognitive development, such as neuroimaging assessments, direct assessments of infant behaviour, IQ, or school performance.

Only studies written in English were included. Randomised control trials (RCTs), case-control trials, and cohort studies were included, whilst case reports, opinion papers, reviews, and study protocols were excluded. Studies of BM supplemented with micronutrients were excluded, as were studies including infants that were formula- or mixed-fed at the time of maternal lactational supplementation or BM micronutrient analysis, as this would compromise measurement of the relationship between BM micronutrients and infant developmental outcomes.

### 2.3. Information Sources

Searches were conducted across the following six databases on the 22nd of February 2021: Medline (Ovid), Embase (Ovid), Global Health (Ovid), PsycINFO (Ovid), Web of Science, and Open Grey. Open Grey was searched for unpublished data.

### 2.4. Search

The search strategy was constructed from a combination of Medical Subject Heading (MeSH) terms, and free text searching of the title and abstract. The following is an example of the search formula input into Medline:

(Infant OR ‘Infant, Newborn’ OR Child OR ‘Child, Preschool’ OR Baby OR Babies OR Neonate) AND (‘Breastfeeding’ OR Lactation OR ‘Milk, human’ OR ‘BM’ OR ‘Human milk’ OR Breastfed OR ‘Exclusive breastfeeding’) AND (Micronutrients OR ‘Vitamin B 6’ OR ‘Vitamin B 6 Deficiency’ OR ‘Vitamin B 12’ OR ‘Vitamin B 12 Deficiency’ OR ‘Vitamin B’ OR Cobalamin OR ‘B-12, Vitamin’ OR Cyanocobalamin OR ‘Vitamin A’ OR ‘Vitamin A Deficiency’ OR Retinol OR ‘All Trans Retinol’ OR ‘Vitamin K’ ‘Vitamin K deficiency’ OR ‘Vitamin E’ OR ‘Vitamin E Deficiency’ OR ‘Vitamin D’ OR ‘Vitamin D Deficiency’ OR Cholecalciferol OR Ergocalciferols OR ‘25-Hydroxyvitamin D 2’ OR ‘Ascorbic acid’ OR ‘Ascorbic acid deficiency’ OR ‘Vitamin C’ OR ‘Vitamin C Deficient’ OR ‘Folic acid’ OR ‘Folic acid deficiency’ OR Folate OR ‘Vitamin B9’ OR Thiamine OR ‘Thiamine deficiency’ OR Thiamin OR ‘Vitamin B1’ OR Riboflavin OR ‘Riboflavin deficiency’ OR ‘Vitamin G’ OR ‘Vitamin B2’ OR Choline OR ‘Choline Deficiency’ OR Iron OR ‘Anemia, Iron deficiency’ OR ‘Ferric compounds’ OR ‘Ferrous compounds’ OR Copper OR Zinc OR Calcium OR ‘Phosphorus compounds’ OR Phosphates OR Magnesium OR ‘Magnesium Deficiency’ OR Selenium OR Iodine OR Iodides) AND (Cognition OR ‘Cognitive function’ OR Learning OR Language OR ‘Language development’ OR ‘Language acquisition’ OR ‘Psychomotor performance’ OR ‘Visual Motor Coordination’ OR ‘Perceptual Motor Performance’ OR ‘Sensory Motor Performance’ OR ‘Neurobehavioral manifestations’ OR ‘Cognitive symptoms’ OR Behavior OR Memory OR Attention OR Emotion OR Bayley OR Socio-emotional OR Sensorimotor OR Intelligence OR IQ OR fMRI OR Electroencephalogram).

The full search strategy can be accessed via this link: https://www.crd.york.ac.uk/PROSPEROFILES/238435_STRATEGY_20210220.pdf (accessed on 15 September 2021), or viewed in the Supplementary Material (Table S2).

### 2.5. Study Selection and the Data Extraction Process

Two reviewers (FL and SMc) independently screened the studies to be included in the review by both title and abstract, according to the above eligibility criteria. Following the initial screening, reviewers discussed their findings to resolve any discrepancies between studies retrieved. Selected studies were recorded using EndNote^TM^ 20 [36].

The lead reviewer (FL) then extracted the following data domains from the full text of each eligible paper according to the Cochrane Data Collection form [37]: date of the study, study design, setting, sample size, participant characteristics, exclusion and inclusion criteria, method of recruitment, method of BM micronutrient measurement, method of infant neurodevelopmental outcome measurement, time-points when BM micronutrients and infant developmental outcomes were measured, study results, and conclusions. This information was input into an Excel spreadsheet and can be viewed in the Supplementary Material (Table S3).

### 2.6. Risk of Bias and Synthesis of Results

The quality of studies was assessed at a study level, according to the Critical Appraisal Skills Programme (CASP) criteria [38]. This included the evaluation of factors such as the study recruitment method and the risk of bias in outcome and exposure measurements [38]. Results of the risk of bias assessment can be viewed in Table S4 of the Supplementary Materials.

Studies were grouped by the micronutrient they investigated, then according to whether methods were observational or evaluated an intervention, and qualitatively synthesised.

## 3. Results

### 3.1. Study Selection

**Figure 1:**
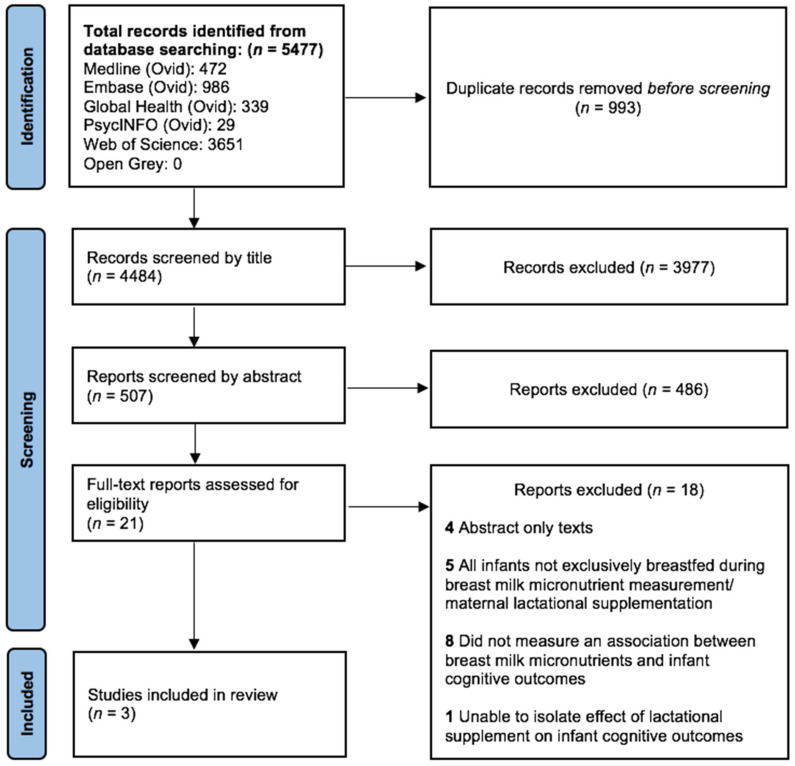
PRISMA flowchart of study selection.

The search generated a total of 5477 results, of which 993 duplicates were removed. After screening by title and abstract, 4463 papers were found to be ineligible. The full texts of the final 21 papers were then examined, as demonstrated below in Figure 1.

Of the remaining records, four were only published as abstracts [39,40,41,42], and therefore could not be included in this review. Following the full-text screening of the other papers, a further five studies were removed [43,44,45,46,47], because all infant participants were not EBF at the time of BM micronutrient measurement [44,45,46,47] or during supplementation [48]. Eight studies were eliminated because they did not explicitly measure (i) BM micronutrients, (ii) infant neurocognitive outcomes, or (iii) an association between these factors [49,50,51,52,53,54,55,56]. One RCT was also excluded because it was not possible to isolate the association between the lactational supplement and the measurement of infant neurodevelopmental outcomes [57]. The remaining three studies included in the review were observational and were published between 2002 and 2020 [58,59,60]. Each included study differed in terms of infant neurodevelopmental outcome measurement, which BM micronutrient was studied, and the study design used. Due to the limited eligible literature retrieved by the search, and the heterogeneity between study designs and the BM micronutrients investigated by each included paper, a meta-analysis was not possible. Study characteristics and results are described below and are summarised in Table 1.

### 3.2. Study Characteristics

#### 3.2.1. Study Setting and Participant Characteristics

Two of the included studies were conducted in high-income countries (HICs), Italy and Poland [59,60], and the third study did not report a location [58]. Sample size varied from a minimum of 25 mother-infant pairs in one study [58], to 370 mother-infant dyads included in the final analysis of the largest study [60]. All studies recruited mothers via convenience sampling from local hospitals [58,59,60], and one additionally invited participants to enrol through social media advertising [59,61]. Participant characteristics varied between studies; in one study, all participants were of low socioeconomic status [58], whilst the other two studies recruited samples of disproportionately high educational attainment [59,60]. None of the papers reported including mothers at risk of or with known micronutrient deficiencies [58,59,60].

#### 3.2.2. Study Design

Two studies were of a prospective cohort study design [59,60], whilst one was cross-sectional [58]. Of the prospective cohort studies, Zielinska et al. [59] conducted three home visits to 39 lactating mothers and their infants at one, three, and six months postpartum [61]. The average levels of selected carotenoids were measured from BM samples collected at one and three months postpartum, and the psychomotor development of infants was analysed at six months of age [59].

The second cohort study followed 370 children and their mothers from 22 weeks gestation until 40 months after birth. BM levels of selenium were measured at one month postpartum, and the cognitive development of infants was evaluated at 40 months [60].

The third, cross-sectional study measured the concentration of vitamin B6 (pyroxidine) in transition milk at 8–11 days postpartum, on the same day that a behavioural assessment of neonates was conducted [58].

#### 3.2.3. Breast Milk Micronutrient Measurement

All three studies differed in terms of which BM micronutrient was studied, and at what time-point BM was analysed. One study measured the vitamin B6 concentration of transition milk in the form of pyridoxal, pyridoxamine, pyridoxine and other phosphorylated variations, from samples taken 8–11 days following birth [58]. The second examined the concentration of the following carotenoids in BM: β-carotene, lycopene, and lutein and its isomer zeaxanthin, calculating a mean value from the concentrations measured at months one and three postpartum [59]. The third study analysed selenium concentrations in BM at one month postpartum [60].

Additionally, these studies examined BM micronutrient composition via different methods; the concentration of selenium in BM was analysed by inductively coupled mass spectrometry [60], whilst BM vitamin B6 and carotenoid levels were determined through high-performance liquid chromatography [58,59].

#### 3.2.4. Infant Developmental Outcome Measures

Studies assessed infant neurodevelopmental outcomes according to the following validated assessment tools: the third edition of the Bayley Scales of Infant and Toddler Development (BSID-III) [60], the Brazelton Neonatal Behavioural Assessment Scale (NBAS) [58], and the Polish Children Development Scale (DSR) [59]. In all three studies, scales were administered by a trained member of the research team [58,59,60].

The BSID-III is a collection of five scales designed to evaluate the main domains of infant neurodevelopment, such as cognition and language development, between the ages of one and 42 months [62]. In this review, the study using the BSID-III only measured infant developmental outcomes using the Cognitive Subscale of the tool, to generate a cognitive composite score at 40 months [60].

The NBAS is a scale that assesses four areas of infant neurobehavioural function: autonomic regulation, motor organisation, state regulation, and attention or social interaction [63]. This tool is intended for use in infants from birth to two months postpartum and gives an overall impression of how well infants are adapting to their extra-uterine environment [63]. The study included in this review conducted the NBAS in infants at 8–11 days postpartum [58].

The DSR is a collection of tests that analyse the behaviour and motor skills of children between 2–36 months [64]. This can be divided into two main scales, the Performance and Observational Scales, which each consist of 10 and four subscales, respectively [60]. The Observational Scale evaluates the temperament of infants, whilst the Performance Scale measures infant abilities in domains such as perception and memory. In the paper included in this review, infant psychomotor development at six months was measured according to six subscales of the Performance Scale of the DSR, which were deemed most suitable for infants of this age [59]. These included measures of motor skills, perception, speech and language, and social behaviour [59].

### 3.3. Risk of Bias within Studies

The risk of bias within all three studies was high according to the CASP assessment tool criteria, as shown in Table S4 of the Supplementary Materials. All studies used convenience sampling, which may have introduced selection bias into results. Additionally, two of the studies had small sample sizes, consisting of 25 and 39 mother-infant dyads [58,59], compromising the generalisability of findings. Furthermore, the specific demographic of participating groups involved in all three studies limits the extent to which findings can be applied to all breastfeeding individuals. For instance, the above-average educational attainment of mothers participating in the study conducted by Castriotta et al. (2020) reduces the applicability of these results to the influence of BM micronutrients in other populations [60].

All included studies used validated tools to assess infant behaviour [58,59,60]. However, given these were methods of direct observation, observer bias may compromise the internal validity of findings. Moreover, all studies only measured infant neurocognitive outcomes on a single occasion, limiting the extent to which the relationship between infant developmental outcomes and BM micronutrients can be assessed. Additionally, in two studies [58,60], the micronutrient composition of BM was analysed from samples taken at a single time-point, reducing the reliability of these exposure measurements.

Only one of the three eligible studies incorporated important confounding variables, such as birth weight, into a linear regression analysis [59]. The two other included papers measured the relationship between BM micronutrients and infant developmental outcomes using Spearman’s rank correlation coefficient [60], and Pearson correlation coefficient [58], without taking account of confounding variables in analyses, reducing the internal validity of this evidence. Finally, one study reported wide confidence intervals (CI) in the data [59], indicating a lack of precision within the results.

### 3.4. Results of Studies

#### 3.4.1. Vitamin B6

One small, cross-sectional study identified a significant correlation between the concentration of pyridoxal in transition milk, and infant scores on two subscales of the NBAS at 8–11 days postpartum (*n* = 25) [58].

The “Habituation” subscale, which evaluates the responses of infants to aversive stimuli [63], was positively correlated with BM pyridoxal concentrations (r = 0.94; *p* ≤ 0.05) [58]. This scale indicates the ability of infants to screen out negative or distracting conditions in their surrounding environment [63].

Additionally, the “Autonomic Stability” scale, which denotes the capacity of infants to control and adjust the CNS, was mildly positively associated with BM pyroxidal content (r = 0.34; *p* ≤ 0.05) [58]. This measurement provides insight into the function and development of neurological homeostatic mechanisms of the neonate [63].

#### 3.4.2. Carotenoids

In a small, prospective cohort study, linear regression revealed that the average BM concentrations of two types of carotenoids, β-carotene and lycopene, from measurements taken at one and three months of lactation were significantly associated with measures of infant psychomotor development at six months (*n* = 39) [59].

The study identified that BM β-carotene was associated with infant scores on the “Motor Development” component of the DSR (β = 0.348; *p* ≤ 0.05 (95% CI 0.036–0.660)) [59], which describes the performance of infants in measurements of fine and gross motor skills, and motility [64]. This relationship remained significant following adjustment for a range of confounding variables, such as infant age, and maternal education (β = 0.296; *p* ≤ 0.05 (95% CI −0.031–0.623)), and when adjusted for infant birth weight and the number of children in the household (β = 0.359; *p* ≤ 0.05 (CI 0.025–0.693)) [59].

Additionally, the concentration of lycopene in BM was significantly associated with unadjusted scores on the DSR “Manipulation” subscale (β = 0.348; *p* ≤ 0.05 (95% CI 0.036–0.660)) [59], which measures the fine motor ability of infants [64]. There was no significant association between the overall DSR Performance Scale scores of infants and any of the BM carotenoids measured [59].

#### 3.4.3. Selenium

In one large, prospective cohort study, a trend level association (*r_s_* = −0.09; *p* = 0.07) was identified between the concentration of selenium in BM at one month postpartum, and cognitive composite scores of infants measured by the BSID-III at 40 months (*n* = 370) [60], however this did not reach statistical significance.

## 4. Discussion

This review aimed to synthesise the available evidence linking micronutrient composition of BM and the developmental outcomes of EBF infants. A systematic search of the literature retrieved only three observational studies eligible for inclusion, demonstrating the scarcity of research conducted in this area. The available studies were highly heterogeneous, and each evaluated a different BM micronutrient [58,59,60], preventing a quantitative synthesis of the data.

Included studies are suggestive of relationships between some BM micronutrients (β-carotene, lycopene [59], and pyroxidal [58]) and infant developmental outcomes. However, considering the low number of papers identified, the observational nature of the studies included, and the high risk of bias within each, this limited evidence is insufficient to establish associations between BM micronutrients and infant neurodevelopment, even for the three micronutrients investigated. Only one included paper continued to follow-up infant participants beyond two years postpartum [60], revealing a gap in literature pertaining to the role of BM micronutrients in infant neurodevelopmental outcomes throughout the first 1000 days. Additionally, whilst carotenoids, vitamin B6, and selenium have important roles in infant neurodevelopment, they represent only a few of the micronutrients and other bioactive components in BM which can influence brain formation and function [8,27]. The influence of many other micronutrients in BM on infant developmental outcomes remains unstudied, raising the question of their significance in postnatal development. Further, none of the eligible research was conducted among populations at high risk of micronutrient deficiencies [58,59,60], limiting the relevance of this research to EBF infants most at risk of micronutrient deficiency. Therefore, this review highlights the lack of evidence available to inform practice and policy to optimise the developmental outcomes of EBF infants.

Of currently published literature, this is the first registered systematic review to examine the association between BM micronutrients and infant neurocognitive outcomes. Two previous Cochrane Reviews sought studies investigating the influence of multiple micronutrient and vitamin A supplementation during lactation [65,66], on measures of infant morbidity. However, neither focused on infant neurodevelopment specifically, or retrieved literature relating micronutrient supplementation during lactation to infant neurocognitive outcomes. Both reviews reported sparse literature on supplementation during lactation and their outcomes of interest, suggesting that the lack of research into the role of breast milk micronutrients identified within this review extends to other outcomes beyond neurocognitive development [65,66].

This research gap may be attributed to the focus of literature on the role of BM macronutrients in infant neurocognitive outcomes [25]. For example, the supplementation of mothers with DHA during breastfeeding is associated with infants achieving improved psychomotor development scores according to the BSID [67]. Recent evidence has also implicated the BM oligosaccharide 2′-fucosyllactose in infant brain development, as one study identified a link between concentrations in early BM and infant cognitive performance at two years of age [68]. Furthermore, the importance of BM composition in early life may be underestimated compared to other factors known to influence neurodevelopment during this critical period [69]. For instance, a considerable amount of research has focused on the role of postnatal iron and iodine deficiency [1,70], stunting [71], and cognitive stimulation in early neurocognitive development [70]. Finally, this paucity of research may reflect the interpretation of the WHO recommendations with the assumption that exclusive breastfeeding universally provides both adequate macro- and micronutrients to support the development of infants under six months of age [29]. This guidance was largely based on EBF infant morbidity and mortality outcomes [29,72], and therefore overlooks potential disparities in neurocognitive development between EBF infants. Additionally, at the time that the WHO policy was published, it was acknowledged that limited data was available on BM micronutrient content and adequacy in relation to infant outcomes, particularly from undernourished groups [73]. It is therefore critical to generate further evidence to ensure exclusive breastfeeding benefits all infants equally.

Evidence indicates that exclusive breastfeeding is beneficial for cognitive development [17,74], an effect that has been identified to persist into adulthood [75], although the majority of this research has been conducted in HICs [74], limiting the generalisability of this data to populations in LMICs. The processes underpinning this association are yet to be fully understood, however, depending on the mechanism, this may or may not be a universal effect. For instance, one potential mechanism linking exclusive breastfeeding and improved developmental outcomes is the impact of prolonged maternal-infant engagement among breastfed infants, compared to those that are formula-fed [76]. The micronutrient composition of breastmilk is another possible mechanism, however, considering the variability in BM micronutrient composition across different populations [30], this is one mechanism that may not be ubiquitous. Therefore, it remains possible that EBF infants of undernourished mothers would not receive the developmental benefits of exclusive breastfeeding policy to the same extent as other infants [8]. This is especially pertinent for infants at high risk of micronutrient deficiency, for example within impoverished or food insecure populations [7]. Ultimately, this adds further vulnerability to infants within these settings where other risk factors are also common.

Overall, this review illustrates that little is understood about the role of BM micronutrient composition in the relationship between exclusive breastfeeding and infant neurocognitive development. Whilst literature recognises the importance of adequate maternal nutrition during exclusive breastfeeding [72,73], the evidence to inform ways to support this in practice is insufficient [66,67]. Furthermore, research in this field is highly skewed towards populations residing in HICs [74], exacerbating the lack of knowledge surrounding the importance of BM micronutrients in the neurocognitive development of EBF infants in LMICs and among populations at greater risk from micronutrient deficiencies. It is essential to ensure that policies and feeding practices are beneficial to all, not just infants in HICs where most of this research has taken place. Considering the high micronutrient requirements of infants during neurodevelopment, and evidence indicating the potentially lifelong impact of nutrition in the first 1000 days of life [2], this represents an area of importance for future research in the interest of population health outcomes globally.

### 4.1. Strengths and Limitations

This review is the first to assess evidence for an association between BM micronutrients and infant neurodevelopmental outcomes. Methodologically, this review was conducted thoroughly; two independent reviewers screened results by title and abstract, ensuring all available evidence was retrieved. The search strategy was inputted into six different databases, with no limitations on dates, geographical region, or age at outcome measurement, increasing the diversity of studies retrieved. Additionally, a broad range of infant developmental outcomes was sought, providing a comprehensive reflection of the available literature. Finally, the inclusion of observational studies enriched the scope of data generated.

However, the design of this review is limited by the strict exclusion criteria used to screen papers. Limiting results to studies published in English may have removed important findings from the search. Further, excluding studies that included mixed-fed infants, breastfed infants supplemented directly with multiple micronutrients, or infants receiving fortified breastmilk, limited the range of papers included in the final analysis. Whilst this was purposeful to increase the ability to assess the association between BM micronutrients and infant developmental outcomes from studies, it resulted in the exclusion of a selection of studies investigating the significance of other BM micronutrients, which may have enriched the findings of this review [43,44,45,46,47].

Limitations of the eligible literature include the high risk of bias across all included studies, restricting the ability to draw conclusions from this evidence. Each eligible study was observational, preventing the assessment of a causal relationship between BM micronutrients and infant developmental outcomes. Additionally, micronutrient availability in early infancy is one of many factors likely to impact development, most of which were not included in analyses [58,59,60], reducing the internal validity of findings. Further, as micronutrient deficiencies often coincide [7], and nutritional deficiencies tend to overlap with other indices of deprivation, it is difficult to disentangle the impact of distinct micronutrients on infant neurodevelopment from these observational studies or to separate the effects from those of other bioactive components in breast milk. Also, all included studies only measured developmental outcomes from a single time-point [58,59,60], limiting the assessment of the possible longitudinal association between BM micronutrients and neurocognitive outcomes.

The generalisability of findings from the eligible literature is compromised by the small sample sizes of two of the included papers [58,59], and the specific demographics of participants in all three studies [58,59,60]. Moreover, all eligible reports only included healthy, term infants [58,59,60], raising the question of the importance of BM micronutrients in the neurodevelopment of infants with differing health needs. The applicability of identified evidence is further restricted by most of the studies being conducted in HICs [59,60]. Finally, none of the eligible research included participants at risk of, or with diagnosed micronutrient deficiencies [58,59,60], limiting the relevance of results to EBF infants most vulnerable to micronutrient deficiencies.

### 4.2. Future Directions

Further research is essential to establish the importance of all of the vitamins and minerals contained in human milk for infant neurodevelopment. Priority micronutrients for future studies include choline, iodine, zinc, vitamin A, vitamin B1, vitamin B2, vitamin B12, and vitamin D, due to their established association with infant brain development [8], close association with maternal status, and the reliance of infants on BM as a postnatal supply [28]. Research should also have a renewed focus on the influence of multiple BM micronutrients on infant neurodevelopment simultaneously since micronutrient deficiencies tend to co-occur [7], and evidence suggests that the concentration of some micronutrients in BM, such as trace elements, may interact with the presence of others [77].

Considering nutrition in early development can have a prolonged influence on neurocognitive function [2], studies should establish whether there is a persisting association between BM micronutrient composition and developmental outcomes, by including long term follow up spanning infancy, childhood and adolescence. This would also allow assessment of different measures of cognitive function which cannot be investigated in infants, such as IQ or socio-emotional development, reflecting the complexity of neurodevelopment more robustly.

Additionally, research should be focused within populations at higher risk of micronutrient deficiency and poor developmental outcomes, to generate findings of higher relevance to policy recommendations relevant for these communities. This would also increase the sensitivity of studies in detecting possible relationships between BM micronutrient composition and infant neurocognitive function.

Examination of the role of BM micronutrients in the developmental outcomes of infants with different health needs is also warranted, for example, among preterm infants. This is especially significant since the concentration of some micronutrients, such as choline and vitamin C, can differ in preterm BM compared to term milk [27]. Although preterm infants may have higher nutritional needs than can be provided exclusively by BM alone, providing supplementary micronutrients alongside BM offers one way to study the influence of these micronutrients on infant neurodevelopment [78,79].

Finally, this field would benefit from additional intervention studies, for instance, examining the influence of maternal micronutrient supplementation during pregnancy and lactation on EBF infant neurocognitive outcomes. This would offer insight into the role of BM micronutrients in infant neurodevelopment, and help to ascertain the safety and efficacy of this potential intervention for EBF infants at risk of micronutrient deficiency. Further, research assessing the effect of the direct supplementation of EBF infants on neurodevelopment is warranted [34], to determine other possible measures to support exclusive breastfeeding in populations at risk of micronutrient deficiency. This is essential considering the first 1000 days of life is a window of opportunity in which nutritional interventions can be most effective at optimising developmental outcomes [2,3].

## 5. Conclusions

In summary, this review retrieved only a small number of observational studies that examined the relationship between selected breast milk micronutrients (vitamin B6, carotenoids, and selenium) and infant neurocognitive outcomes. Considering the low number and quality of studies, these findings are inadequate to establish relationships between breast milk micronutrients and infant neurocognitive outcomes. Further study is required to inform policy recommendations and practice and maximise the benefits of exclusive breastfeeding for the neurocognitive development of all infants.

## Figures and Tables

**Table 1:** The study design and main results of each included study (*n* = 3).

Study ID	Number of Participants	Population Characteristics	Breast Milk Micronutrient Measured	Time-Point of Breast Milk Micronutrient Measurement	Infant Developmental Outcome Measure	Time-Point of Infant Developmental Outcome Measurement	Main Results
Cross-Sectional Study Design							
Boylan et al., 2002 [58]	25	All participants were from low-income backgrounds	Vitamin B6 in the form of pyridoxal, pyridoxamine, and pyridoxine	8–11 days postpartum	Brazelton Neonatal Behavioural Assessment Scale (NBAS)	8–11 days postpartum	A significant, positive correlation was identified between breast milk pyroxidal concentration and the Habituation subscale (r = 0.94; *p* ≤ 0.05), and the Autonomic Stability subscale of the NBAS (r = 0.34; *p* ≤ 0.05).
**Prospective Cohort Study Design**							
Zielinska et al., 2019a [59]	39	Mothers had a higher than average educational level and high average income	The carotenoids β-carotene, lycopene, and lutein and zeaxanthin	One and three months postpartum	Six sub-scales of the Polish Children Development Scale (DSR): Manipulation, Perception, Memory, Speech and language, Social behaviour, and Motor skills	Six months postpartum	Breast milk β-carotene was significantly associated with infant scores on the Motor Development subscale of the DSR (β = 0.348; *p* ≤ 0.05 (95% CI 0.036–0.660)). This association remained significant following adjustment for confounding variables such as infant age, and maternal education (β = 0.296; *p* ≤ 0.05 (95% CI −0.031–0.623)), and following adjustment for infant birth weight and the number of children in the household (β = 0.359; *p* ≤ 0.05 (CI 0.025–0.693)). Breast milk lycopene was significantly associated with infant unadjusted scores on the Manipulation subscale of the DSR (β = 0.348; *p* ≤ 0.05 (95% CI 0.036–0.660)).
Castriotta et al., 2020 [60]	370	Most mothers had a college degree, and the average maternal non-verbal intelligence score was high	Selenium	One month postpartum	The Cognitive Scale of the third edition of the Bayley Scales of Infant and Toddler Development (BSID-III)	40 months postpartum	A trend level association was identified between the concentration of selenium in breast milk and the cognitive composite scores of infants on the BSID-III (*r_s_* = −0.09; *p* = 0.07), however this did not reach statistical significance.

## Data Availability

Not applicable.

## Supplementary Materials

The following are available online at https://www.mdpi.com/article/10.3390/nu13113848/s1, Table S1: PRISMA 2020 Checklist, Table S2: Search strategy, Table S3: Data extraction table, Table S4: Risk of bias assessment.
