# Supplementary material for: Breast Milk Micronutrients and Infant Neurodevelopmental Outcomes: A Systematic Review
Source: Nutrients. 2021 Oct 28;13(11):3848. doi: 10.3390/nu13113848 (PMC8624933; doi:10.3390/nu13113848)
Supplement: Supplementary file 1 [file nutrients-13-03848-s001.zip › nutrients-1411975-supplementary.pdf]

Table S1: PRISMA 2020 Checklist

Section and Topic	Item #	Checklist item	Reported on page #
**TITLE**			
Title	1	Identify the report as a systematic review.	1
**ABSTRACT**			
Abstract	2	See the PRISMA 2020 for Abstracts checklist.	1
**INTRODUCTION**			
Rationale	3	Describe the rationale for the review in the context of existing knowledge.	1-3
Objectives	4	Provide an explicit statement of the objective(s) or question(s) the review addresses.	3
**METHODS**			
Eligibility criteria	5	Specify the inclusion and exclusion criteria for the review and how studies were grouped for the syntheses.	3-4
Information sources	6	Specify all databases, registers, websites, organisations, reference lists and other sources searched or consulted to identify studies. Specify the date when each source was last searched or consulted.	4
Search strategy	7	Present the full search strategies for all databases, registers and websites, including any filters and limits used.	5
Selection process	8	Specify the methods used to decide whether a study met the inclusion criteria of the review, including how many reviewers screened each record and each report retrieved, whether they worked independently, and if applicable, details of automation tools used in the process.	4
Data collection process	9	Specify the methods used to collect data from reports, including how many reviewers collected data from each report, whether they worked independently, any processes for obtaining or confirming data from study investigators, and if applicable, details of automation tools used in the process.	4
Data items	10a	List and define all outcomes for which data were sought. Specify whether all results that were compatible with each outcome domain in each study were sought (e.g. for all measures, time points, analyses), and if not, the methods used to decide which results to collect.	4
	10b	List and define all other variables for which data were sought (e.g. participant and intervention characteristics, funding sources). Describe any assumptions made about any missing or unclear information.	4
Study risk of bias assessment	11	Specify the methods used to assess risk of bias in the included studies, including details of the tool(s) used, how many reviewers assessed each study and whether they worked independently, and if applicable, details of automation tools used in the process.	4
Effect measures	12	Specify for each outcome the effect measure(s) (e.g. risk ratio, mean difference) used in the synthesis or presentation of results.	N/A
Synthesis methods	13a	Describe the processes used to decide which studies were eligible for each synthesis (e.g. tabulating the study intervention characteristics and comparing against the planned groups for each synthesis (item #5)).	N/A
	13b	Describe any methods required to prepare the data for presentation or synthesis, such as handling of missing summary statistics, or data conversions.	N/A
	13c	Describe any methods used to tabulate or visually display results of individual studies and syntheses.	N/A

Section and Topic	Item #	Checklist item	Reported on page #
	13d	Describe any methods used to synthesize results and provide a rationale for the choice(s). If meta-analysis was performed, describe the model(s), method(s) to identify the presence and extent of statistical heterogeneity, and software package(s) used.	7
	13e	Describe any methods used to explore possible causes of heterogeneity among study results (e.g. subgroup analysis, meta-regression).	N/A
	13f	Describe any sensitivity analyses conducted to assess robustness of the synthesized results.	N/A
Reporting bias assessment	14	Describe any methods used to assess risk of bias due to missing results in a synthesis (arising from reporting biases).	N/A
Certainty assessment	15	Describe any methods used to assess certainty (or confidence) in the body of evidence for an outcome.	N/A
**RESULTS**			
Study selection	16a	Describe the results of the search and selection process, from the number of records identified in the search to the number of studies included in the review, ideally using a flow diagram.	5-6
	16b	Cite studies that might appear to meet the inclusion criteria, but which were excluded, and explain why they were excluded.	6
Study characteristics	17	Cite each included study and present its characteristics.	7-8
Risk of bias in studies	18	Present assessments of risk of bias for each included study.	8
Results of individual studies	19	For all outcomes, present, for each study: (a) summary statistics for each group (where appropriate) and (b) an effect estimate and its precision (e.g. confidence/credible interval), ideally using structured tables or plots.	N/A
Results of syntheses	20a	For each synthesis, briefly summarise the characteristics and risk of bias among contributing studies.	N/A
	20b	Present results of all statistical syntheses conducted. If meta-analysis was done, present for each the summary estimate and its precision (e.g. confidence/credible interval) and measures of statistical heterogeneity. If comparing groups, describe the direction of the effect.	N/A
	20c	Present results of all investigations of possible causes of heterogeneity among study results.	N/A
	20d	Present results of all sensitivity analyses conducted to assess the robustness of the synthesized results.	N/A
Reporting biases	21	Present assessments of risk of bias due to missing results (arising from reporting biases) for each synthesis assessed.	N/A
Certainty of evidence	22	Present assessments of certainty (or confidence) in the body of evidence for each outcome assessed.	N/A
**DISCUSSION**			
Discussion	23a	Provide a general interpretation of the results in the context of other evidence.	11-12
	23b	Discuss any limitations of the evidence included in the review.	13
	23c	Discuss any limitations of the review processes used.	12-13
	23d	Discuss implications of the results for practice, policy, and future research.	11-14

Section and Topic	Item #	Checklist item	Reported on page #
**OTHER INFORMATION**			
Registration and protocol	24a	Provide registration information for the review, including register name and registration number, or state that the review was not registered.	3
	24b	Indicate where the review protocol can be accessed, or state that a protocol was not prepared.	3
	24c	Describe and explain any amendments to information provided at registration or in the protocol.	N/A
Support	25	Describe sources of financial or non-financial support for the review, and the role of the funders or sponsors in the review.	14
Competing interests	26	Declare any competing interests of review authors.	14
Availability of data, code and other materials	27	Report which of the following are publicly available and where they can be found: template data collection forms; data extracted from included studies; data used for all analyses; analytic code; any other materials used in the review.	14

Table S2: Search strategy

Database	Search terms
Medline (Ovid)	(Infant OR 'Infant, Newborn' OR Child OR 'Child, Preschool' OR Baby OR Babies OR Neonate) AND ('Breast Feeding' OR Lactation OR 'Milk, human' OR 'Breast milk' OR 'Human milk' OR Breastfed OR 'Exclusive breastfeeding') AND (Micronutrients OR 'Vitamin B 6' OR 'Vitamin B 6 Deficiency' OR 'Vitamin B 12' OR 'Vitamin B 12 Deficiency' OR 'Vitamin B' OR Cobalamin OR 'B-12, Vitamin' OR Cyanocobalamin OR 'Vitamin A' OR 'Vitamin A Deficiency' OR Retinol OR 'All Trans Retinol' OR 'Vitamin K' 'Vitamin K deficiency' OR 'Vitamin E' OR 'Vitamin E Deficiency' OR 'Vitamin D' OR 'Vitamin D Deficiency' OR Cholecalciferol OR Ergocalciferols OR '25-Hydroxyvitamin D 2' OR 'Ascorbic acid' OR 'Ascorbic acid deficiency' OR 'Vitamin C' OR 'Vitamin C Deficient' OR 'Folic acid' OR 'Folic acid deficiency' OR Folate OR 'Vitamin B9' OR Thiamine OR 'Thiamine deficiency' OR Thiamin OR 'Vitamin B1' OR Riboflavin OR 'Riboflavin deficiency' OR 'Vitamin G' OR 'Vitamin B2' OR Choline OR 'Choline Deficiency' OR Iron OR 'Anemia, Iron deficiency' OR 'Ferric compounds' OR 'Ferrous compounds' OR Copper OR Zinc OR Calcium OR 'Phosphorus compounds' OR Phosphates OR Magnesium OR 'Magnesium Deficiency' OR Selenium OR Iodine OR Iodides) AND (Cognition OR 'Cognitive function' OR Learning OR Language OR 'Language development' OR 'Language acquisition' OR 'Psychomotor performance' OR 'Visual Motor Coordination' OR 'Perceptual Motor Performance' OR 'Sensory Motor Performance' OR 'Neurobehavioral manifestations' OR 'Cognitive symptoms' OR Behavior OR Memory OR Attention OR Emotion OR Bayley OR Socio-emotional OR Sensorimotor OR Intelligence OR IQ OR fMRI OR Electroencephalogram)
Embase (Ovid)	(Infant OR 'Infant, Newborn' OR Child OR 'Child, Preschool' OR Baby OR Babies OR Neonate) AND ('Breast Feeding' OR Lactation OR 'Milk, human' OR 'Breast milk' OR 'Human milk' OR Breastfed OR 'Exclusive breastfeeding') AND (Micronutrients OR 'Vitamin B 6' OR 'Vitamin B 6 Deficiency' OR 'Vitamin B 12' OR 'Vitamin B 12 Deficiency' OR 'Vitamin B' OR Cobalamin OR 'B-12, Vitamin' OR Cyanocobalamin OR 'Vitamin A' OR 'Vitamin A Deficiency' OR Retinol OR 'All Trans Retinol' OR 'Vitamin K' 'Vitamin K deficiency' OR 'Vitamin E' OR 'Vitamin E Deficiency' OR 'Vitamin D' OR 'Vitamin D Deficiency' OR Cholecalciferol OR Ergocalciferols OR '25-Hydroxyvitamin D 2' OR 'Ascorbic acid' OR 'Ascorbic acid deficiency' OR 'Vitamin C' OR 'Vitamin C Deficient' OR 'Folic acid' OR 'Folic acid deficiency' OR Folate OR 'Vitamin B9' OR Thiamine OR 'Thiamine deficiency' OR Thiamin OR 'Vitamin B1' OR Riboflavin OR 'Riboflavin deficiency' OR 'Vitamin G' OR 'Vitamin B2' OR Choline OR 'Choline Deficiency' OR Iron OR 'Anemia, Iron deficiency' OR 'Ferric compounds' OR 'Ferrous compounds' OR Copper OR Zinc OR Calcium OR 'Phosphorus compounds' OR Phosphates OR Magnesium OR 'Magnesium Deficiency' OR Selenium OR Iodine OR

	Iodides) AND (Cognition OR 'Cognitive function' OR Learning OR Language OR 'Language development' OR 'Language acquisition' OR 'Psychomotor performance' OR 'Visual Motor Coordination' OR 'Perceptual Motor Performance' OR 'Sensory Motor Performance' OR 'Neurobehavioral manifestations' OR 'Cognitive symptoms' OR Behavior OR Memory OR Attention OR Emotion OR Bayley OR Socio-emotional OR Sensorimotor OR Intelligence OR IQ OR fMRI OR Electroencephalogram)
PsycInfo (Ovid)	(Infant OR 'Infant, Newborn' OR Child OR 'Child, Preschool' OR Baby OR Babies OR Neonate) AND ('Breast Feeding' OR Lactation OR 'Milk, human' OR 'Breast milk' OR 'Human milk' OR Breastfed OR 'Exclusive breastfeeding') AND (Micronutrients OR 'Vitamin B 6' OR 'Vitamin B 6 Deficiency' OR 'Vitamin B 12' OR 'Vitamin B 12 Deficiency' OR 'Vitamin B' OR Cobalamin OR 'B-12, Vitamin' OR Cyanocobalamin OR 'Vitamin A' OR 'Vitamin A Deficiency' OR Retinol OR 'All Trans Retinol' OR 'Vitamin K' 'Vitamin K deficiency' OR 'Vitamin E' OR 'Vitamin E Deficiency' OR 'Vitamin D' OR 'Vitamin D Deficiency' OR Cholecalciferol OR Ergocalciferols OR '25-Hydroxyvitamin D 2' OR 'Ascorbic acid' OR 'Ascorbic acid deficiency' OR 'Vitamin C' OR 'Vitamin C Deficient' OR 'Folic acid' OR 'Folic acid deficiency' OR Folate OR 'Vitamin B9' OR Thiamine OR 'Thiamine deficiency' OR Thiamin OR 'Vitamin B1' OR Riboflavin OR 'Riboflavin deficiency' OR 'Vitamin G' OR 'Vitamin B2' OR Choline OR 'Choline Deficiency' OR Iron OR 'Anemia, Iron deficiency' OR 'Ferric compounds' OR 'Ferrous compounds' OR Copper OR Zinc OR Calcium OR 'Phosphorus compounds' OR Phosphates OR Magnesium OR 'Magnesium Deficiency' OR Selenium OR Iodine OR Iodides) AND (Cognition OR 'Cognitive function' OR Learning OR Language OR 'Language development' OR 'Language acquisition' OR 'Psychomotor performance' OR 'Visual Motor Coordination' OR 'Perceptual Motor Performance' OR 'Sensory Motor Performance' OR 'Neurobehavioral manifestations' OR 'Cognitive symptoms' OR Behavior OR Memory OR Attention OR Emotion OR Bayley OR Socio-emotional OR Sensorimotor OR Intelligence OR IQ OR fMRI OR Electroencephalogram)
Global Health (Ovid)	(Infant OR 'Infant, Newborn' OR Child OR 'Child, Preschool' OR Baby OR Babies OR Neonate) AND ('Breast Feeding' OR Lactation OR 'Milk, human' OR 'Breast milk' OR 'Human milk' OR Breastfed OR 'Exclusive breastfeeding') AND (Micronutrients OR 'Vitamin B 6' OR 'Vitamin B 6 Deficiency' OR 'Vitamin B 12' OR 'Vitamin B 12 Deficiency' OR 'Vitamin B' OR Cobalamin OR 'B-12, Vitamin' OR Cyanocobalamin OR 'Vitamin A' OR 'Vitamin A Deficiency' OR Retinol OR 'All Trans Retinol' OR 'Vitamin K' 'Vitamin K deficiency' OR 'Vitamin E' OR 'Vitamin E Deficiency' OR 'Vitamin D' OR 'Vitamin D Deficiency' OR Cholecalciferol OR Ergocalciferols OR '25-Hydroxyvitamin D 2' OR 'Ascorbic acid' OR 'Ascorbic acid deficiency' OR 'Vitamin C' OR 'Vitamin C Deficient' OR 'Folic acid' OR 'Folic acid deficiency' OR Folate OR 'Vitamin B9' OR Thiamine OR 'Thiamine deficiency' OR Thiamin OR 'Vitamin B1' OR Riboflavin OR 'Riboflavin deficiency' OR 'Vitamin G' OR 'Vitamin B2' OR Choline OR 'Choline Deficiency' OR Iron OR 'Anemia, Iron deficiency' OR 'Ferric compounds' OR 'Ferrous compounds' OR Copper OR Zinc OR Calcium OR 'Phosphorus compounds' OR Phosphates OR Magnesium OR 'Magnesium Deficiency' OR Selenium OR Iodine OR

	Iodides) AND (Cognition OR 'Cognitive function' OR Learning OR Language OR 'Language development' OR 'Language acquisition' OR 'Psychomotor performance' OR 'Visual Motor Coordination' OR 'Perceptual Motor Performance' OR 'Sensory Motor Performance' OR 'Neurobehavioral manifestations' OR 'Cognitive symptoms' OR Behavior OR Memory OR Attention OR Emotion OR Bayley OR Socio-emotional OR Sensorimotor OR Intelligence OR IQ OR fMRI OR Electroencephalogram)
Web of Science	(Infant OR Neonat* OR Child* OR Newborn OR 'Pre-school child*' OR Infancy OR Baby OR Babies) AND ('Breast Feeding' OR Breastfed OR Lactati* OR 'Breast milk' OR 'Human milk' OR 'Exclusive breastfeeding') AND (Micronutrient OR 'Trace Element' OR Vitamin OR 'Vitamin A' OR 'Vitamin A Deficien*' OR Retinol OR 'All Trans Retinol' OR 'Vitamin B-6' OR 'Vitamin B-6 Deficien*' OR 'Vitamin B 12' OR 'Vitamin B 12 Deficien*' OR Cobalamin OR 'B-12, Vitamin' OR Cyanocobalamin OR 'Vitamin K' OR 'Vitamin K Deficien*' OR 'Vitamin E' OR 'Vitamin E Deficien*' OR 'Vitamin D' OR 'Vitamin D Deficien*' OR Cholecalciferol OR Ergocalciferol OR '25-Hydroxyvitamin D' OR 'Ascorbic acid' OR 'Vitamin C' OR 'Vitamin C Deficien*' OR 'Folic acid' OR 'Folic Acid Deficien*' OR Folate OR Thiamin* OR 'Thiamin* Deficien*' OR 'Vitamin B1' OR Riboflavin OR 'Riboflavin Deficien*' OR 'Vitamin G' OR Choline OR 'Choline Deficien*' OR Iron OR 'Iron Deficien*' OR Ferric* OR Ferrous* OR Copper OR 'Copper Deficien*' OR Zinc OR 'Zinc Deficien*' OR Calcium OR 'Calcium Deficien*' OR Phosphorus OR Phosphate OR Magnesium OR 'Magnesium deficiency' OR Selenium OR Iodi*e) AND (Cogniti* OR Neurobehavio*r* OR Motor OR 'Psychomotor performance' OR Language OR Behavi* OR Neurodevelopment* OR Learning OR Memory OR Emoti* OR Bayley OR Socio-emotion* OR Sensorimotor OR Intelligence OR IQ OR fMRI OR Electroencephalogram OR Attention OR 'Cognitive symptoms' OR 'Visual Motor Coordination' OR 'Perceptual Motor Performance' OR 'Sensory Motor Performance' OR 'Language acquisition' OR Language OR 'Language development')
Open Grey	(Infant OR Neonat* OR Child* OR Newborn OR 'Pre-school child*' OR Infancy OR Baby OR Babies) AND ('Breast Feeding' OR Breastfed OR Lactati* OR 'Breast milk' OR 'Human milk' OR 'Exclusive breastfeeding') AND (Micronutrient OR 'Trace Element' OR Vitamin OR 'Vitamin A' OR 'Vitamin A Deficien*' OR Retinol OR 'All Trans Retinol' OR 'Vitamin B-6' OR 'Vitamin B-6 Deficien*' OR 'Vitamin B 12' OR 'Vitamin B 12 Deficien*' OR Cobalamin OR 'B-12, Vitamin' OR Cyanocobalamin OR 'Vitamin K' OR 'Vitamin K Deficien*' OR 'Vitamin E' OR 'Vitamin E Deficien*' OR 'Vitamin D' OR 'Vitamin D Deficien*' OR Cholecalciferol OR Ergocalciferol OR '25-Hydroxyvitamin D' OR 'Ascorbic acid' OR 'Vitamin C' OR 'Vitamin C Deficien*' OR 'Folic acid' OR 'Folic Acid Deficien*' OR Folate OR Thiamin* OR 'Thiamin* Deficien*' OR 'Vitamin B1' OR Riboflavin OR 'Riboflavin Deficien*' OR 'Vitamin G' OR Choline OR 'Choline Deficien*' OR Iron OR 'Iron Deficien*' OR Ferric* OR Ferrous* OR Copper OR 'Copper Deficien*' OR Zinc OR 'Zinc Deficien*' OR Calcium OR 'Calcium Deficien*' OR Phosphorus OR Phosphate OR Magnesium OR 'Magnesium deficiency' OR Selenium OR Iodi*e) AND (Cogniti* OR Neurobehavio*r* OR Motor OR 'Psychomotor

	performance' OR Language OR Behavi* OR Neurodevelopment* OR Learning OR Memory OR Emoti* OR Bayley OR Socio-emotion* OR Sensorimotor OR Intelligence OR IQ OR fMRI OR Electroencephalogram OR Attention OR 'Cognitive symptoms' OR 'Visual Motor Coordination' OR 'Perceptual Motor Performance' OR 'Sensory Motor Performance' OR 'Language acquisition' OR Language OR 'Language development')

Table S3: Data extraction table

Author and date of study	Boylan et al. 2002	Zielinska et al. 2019	Castriotta et al. 2020
**Setting**	Unknown	Poland	Italy
**Recruitment method**	Convenience sampling from a local hospital	Convenience sampling from a local hospital, advertisement on social media groups	Convenience sampling from a local hospital
**Number of participants**	25	39	370
**Inclusion criteria**	Term infants, uncomplicated pregnancies, Apgar scores above seven	Mothers aged over 19, “healthy” infant, infant age below six weeks, singleton birth, mother plans to exclusively breastfeed for six months	Pregnant women aged over 18, permanent residents of the study area for over two years
**Exclusion criteria**	Maternal drug or alcohol abuse, use of prescription medications, current infections, chronic medical conditions	Infants not exclusively breastfed, low birth weight infants, preterm infants, infants with birth defects, maternal chronic medical conditions, pregnancy complications, maternal alcohol or tobacco use, maternal vegan diet, low breast milk supply	Preterm infants, infants with congenital malformations/ severe health problems which would affect neurological development, maternal drug abuse, maternal health problems, pregnancy complications, absence from study area for over six weeks, twin pregnancy
**Participant characteristics**	All participants were from low-income backgrounds.	Most mothers had a university education.<br>Most mothers had a high average income.	Most mothers had a college degree. The average non-verbal intelligence score of mothers was high.<br>Most mothers had maternity leave during pregnancy.

**Study design**	Cross sectional study design. Transition milk samples collected 8-11 days after delivery and analysed for vitamin B-6 content. Neurobehavioural function of infants was determined from the Brazelton Neonatal Behavioural Assessment Scale at the home visit at 8-11 days postpartum when breast milk samples were collected.	Prospective cohort study design. Breast milk samples collected at one, three and six months postpartum which were analysed for carotenoid content. Average carotenoid content of breast milk was calculated from measurements taken at one and three months postpartum. Infant psychomotor development was measured according to the Polish Child Development Scale at six months postpartum.	Prospective cohort study design. Breast milk samples collected and were analysed for selenium at one month postpartum. Infant cognitive function was determined at 40 months postpartum from the Cognitive Scale of the Bayley Scales of Infant and Toddler Development (third edition).
**Measurement of infant neurodevelopmental outcomes**	The Brazelton Neonatal Behavioural Assessment Scale	Six subtests of the Polish Child Development Scale: Manipulation, Perception, Memory, Speech and language, Social behaviour, and Motor skills	The Cognitive Scale of the Bayley Scales of Infant and Toddler Development (third edition)
**Time-point of infant neurodevelopmental outcome measurement**	8-11 days postpartum	Six months postpartum	40 months postpartum
**Breast milk micronutrient measured**	Vitamin B6 in the form of pyridoxal, pyridoxamine, pyridoxine, and other phosphorylated forms	The carotenoids β-carotene, lycopene, and lutein and zeaxanthin	Selenium

**Method of breast milk micronutrient measurement**	High performance liquid chromatography	High performance liquid chromatography	Inductively coupled mass spectrometry
**Time-point of breast milk micronutrient measurement**	8-11 postpartum	One and three months postpartum	One month postpartum
**Results**	Positive correlation between the pyroxidal content of breast milk and infant scores on two NBAS subscales (calculated from Pearson correlation coefficient), the infant Habituation subscale (r=0.94), and Autonomic Stability (r=0.34) subscales.	Breast milk β-carotene was significantly associated with infant motor development (β = 0.348; $p \leq 0.05$ (95% CI 0.036–0.660)). This association remained significant after linear regression was adjusted for infant age, gender, maternal age, education, and maternal psychological status (β = 0.296; $p \leq 0.05$ (95% CI −0.031–0.623)), and after adjustment for birth weight and parity (β = 0.359; $p \leq$ 0.05 (95% CI 0.025–0.693)). Lycopene was significantly associated with the adjusted manipulation subscale scores (β = 0.348; $p \leq 0.05$ (95% CI 0.036-0.660)). There were no significant associations between any breast milk carotenoids and overall scores of infants on the Performance Scale.	Spearmann correlation coefficient of -0.09 between breastmilk selenium and infant composite scores (*p* =0.07).<br>Of infants with suboptimal cognitive composite scores, lower than the first quintile score of 100, (N=75), mean selenium in breast milk was 19.16ng/g, standard deviation 6.05, median 19.21 ng/g, 7.84 inter-quartile range. In infants with composite cognitive scores higher than the first quintile (N=295), the mean selenium in breastmilk was 18.59ng/g, standard deviation 6.21, median 18.06ng/g, inter-quartile range 6.5. Difference between the breast milk selenium exposure of groups was non-significant according to the p value generated by

			Mann Whitney Test (p= 0.45).
**Conclusion**	"Vitamin B6 is important for the normal behavioural function of infants"	"Due to the positive association between concentrations of… β-carotene in breastmilk and infant motor development, it is important to provide these nutrients with breastmilk"	Not reported

Table S4: Risk of bias assessment

Study ID	Boylan et al. 2002	Zielinska et al. 2019	Castriotta et al. 2020
**Risk of selection bias**	High – convenience sampling	High – convenience and volunteer sampling	High – convenience sampling
**Is the cohort representative of all breastfeeding women?**	No – small sample (N=25), all from a low-income background	No – small sample (N=39), most participants had university education and the average participant income was high	No - a large proportion of mothers had a college degree, and the average non-verbal intelligence score was high
**Risk of bias - measurement of exposure to breast milk micronutrients**	High – breast milk vitamin B-6 objectively measured by high performance liquid chromatography, only measured on one occasion at 8-11 days postpartum	Low – breast milk carotenoids objectively measured by high performance liquid chromatography, average of two measurements taken at one and three months postpartum	High – breast milk selenium objectively measured by inductively coupled mass spectrometry is objective, only measured on one occasion at 40 months postpartum

**Risk of bias – infant developmental outcome measurement**	Medium – validated assessment tool (Brazelton Neonatal Behavioural Assessment Scale), conducted by a trained member of the research team, direct behavioural assessment introducing risk of observer bias	Medium - validated assessment tool (the Polish Child Development Scale), conducted by a trained member of the research team, direct behavioural assessment introducing risk of observer bias	Medium – validated assessment tool (Bayley Scales of Infant and Toddler Development (third edition)), conducted by a trained member of the research team, direct behavioural assessment introducing risk of observer bias
**Confounding factors identified by authors**	No	Yes – authors identified the following confounding factors: infant age, gender, maternal age, education, maternal psychological status, infant birth weight, and maternal parity	Yes – authors identified the following confounding factors: BMI before pregnancy, maternal intelligence quotient, mother's occupational status, size of home, maternal smoking and alcohol intake during pregnancy, maternal intake of fish during pregnancy, duration of breastfeeding up to 18 months, child fish consumption

**Confounding factors accounted for in analysis or study design**	No	Yes – two linear regression models were made which adjusted for confounding. The first adjusted for infant age, gender, maternal age, education, maternal psychological status. The second adjusted for birth weight and parity.	No
**Was the follow up of subjects complete enough?**	No – infant neuro-behavioural outcomes only measured on one occasion, at the same time as breast milk vitamin B-6 measurement, measured soon following birth, long term relevance unknown	No – infant psychomotor development only measured on one occasion, only used six subscales of the Polish Child Development Scale, long term relevance unknown	No – infant cognitive outcomes only measured on one occasion, only one subscale (the Cognitive Subscale) of the Bayley Scales of Infant and Toddler Development used, long term relevance unknown
**Precision of the results (confidence intervals)**	Not reported	Low - wide confidence intervals	Not reported
**Overall risk of bias**	High	High	High
